# Supplementary material for: Complexity of frequency receptive fields predicts tonotopic variability across species
Source: eLife. 2020 May 18;9:e53462. doi: 10.7554/eLife.53462 (PMC7269667; doi:10.7554/eLife.53462)
Supplement: Supplementary file 1. [file elife-53462-supp1.docx]

eLife 08-11-2019-RA-eLife-53462

"Complexity of frequency receptive fields predicts tonotopic variability across species"

Gaucher, Panniello et al.

**Supplementary statistics for ANOVAs and t-tests presented in the manuscript**

**1.** **Figure 2C**

Unpaired t-test comparing the local BF variation in single-peaked neurons and in double-peaked neurons in ferrets.

| Group | Mean | Standard deviation | N (FRA) | t value | p value | Effect size  (Cohen’s d) |
| --- | --- | --- | --- | --- | --- | --- |
| Single-peaked | 1.344 | 1.650 | 245 | 4.148 | 4.09x10^-5^ | -0.419 |
| Double-peaked | 2.166 | 2.356 | 163 |  |  |  |

Unpaired t-test comparing the local BF variation in single-peaked neurons and in complex neurons in ferrets.

| Group | Mean | Standard deviation | N (FRA) | t value | p value | Effect size  (Cohen’s d) |
| --- | --- | --- | --- | --- | --- | --- |
| Single-peaked | 1.344 | 1.650 | 245 | 7.547 | 3.39x10^-13^ | -0.813 |
| Complex | 3.052 | 2.748 | 133 |  |  |  |

Paired t-test comparing the local BF variation in double-peaked neurons and in the second peak of double-peaked neurons in ferrets.

| Group | Mean | Standard deviation | N (FRA) | t value | p value | Effect size  (Cohen’s d) |
| --- | --- | --- | --- | --- | --- | --- |
| Double-peaked | 2.166 | 2.356 | 163 | 1.914 | 0.057 | -0.210 |
| Peak2 | 2.695 | 2.670 | 163 |  |  |  |

**2. Figure 2D**

Unpaired t-test comparing the response strength in single-peaked neurons and in double-peaked neurons in ferrets.

| Group | Mean | Standard deviation | N (FRA) | t value | p value | Effect size  (Cohen’s d) |
| --- | --- | --- | --- | --- | --- | --- |
| Single-peaked | 0.905 | 0.809 | 296 | 2.218 | 0.027 | 0.197 |
| Double-peaked | 0.738 | 0.896 | 220 |  |  |  |

Unpaired t-test comparing the response strength in single-peaked neurons and in complex neurons in ferrets.

| Group | Mean | Standard deviation | N (FRA) | t value | p value | Effect size  (Cohen’s d) |
| --- | --- | --- | --- | --- | --- | --- |
| Single-peaked | 0.905 | 0.809 | 296 | 4.015 | 6.9x10^-5^ | 0.197 |
| Complex | 0.604 | 0.753 | 177 |  |  |  |

Unpaired t-test comparing the response strength in double-peaked neurons and in complex neurons in ferrets.

| Group | Mean | Standard deviation | N (FRA) | t value | p value | Effect size  (Cohen’s d) |
| --- | --- | --- | --- | --- | --- | --- |
| Double-peaked | 0.738 | 0.896 | 220 | 1.584 | 0.11 | 0.160 |
| Complex | 0.604 | 0.753 | 177 |  |  |  |

**3. Figure 2E**

Unpaired t-test comparing Fano Factors of neuronal activity in single-peaked neurons and in double-peaked neurons.

| Group | Mean | Standard deviation | N (FRA) | t value | p value | Effect size  (Cohen’s d) |
| --- | --- | --- | --- | --- | --- | --- |
| Single-peaked | 1.547 | 1.957 | 296 | 0.600 | 0.549 | -0.054 |
| Double-peaked | 1.668 | 2.608 | 217 |  |  |  |

Unpaired t-test comparing Fano Factors of neuronal activity in single-peaked neurons and in complex neurons.

| Group | Mean | Standard deviation | N (FRA) | t value | p value | Effect size  (Cohen’s d) |
| --- | --- | --- | --- | --- | --- | --- |
| Single-peaked | 1.547 | 1.957 | 296 | 0.638 | 0.524 | 0.061 |
| Complex | 1.423 | 2.185 | 175 |  |  |  |

**4. Figure 3A**

1-way ANOVA comparing pairwise signal correlations with FRA class as a predictor.

| Dependent variable | Degrees of freedom | F value | p value | Effect size (Eta squared) |
| --- | --- | --- | --- | --- |
| FRA class | 2 | 389.7 | 1.557x10^-163^ | 0.074 |

Post-hoc pairwise comparisons between signal correlations of different FRA types performed on results of the above ANOVA, using Tukey’s HSD criterion.

| Compared FRA types | | Lower confidence interval | Mean | Upper confidence interval | p value |
| --- | --- | --- | --- | --- | --- |
| Single-peaked | Double-peaked | 0.0101 | 0.0219 | 0.0338 | 4.430x10^-5^ |
| Single-peaked | Complex | 0.1928 | 0.2104 | 0.2281 | 9.561x10^-10^ |
| Double-Peaked | Complex | 0.1688 | 0.1885 | 0.2083 | 9.561x10^-10^ |

**5. Figure 3B**

1-way ANOVA comparing pairwise noise correlations with FRA class as a predictor in ferret.

| Dependent variable | Degrees of freedom | F value | p value | Effect size (Eta squared) |
| --- | --- | --- | --- | --- |
| FRA class | 2 | 109.5 | 9.405x10^-48^ | 0.022 |

Post-hoc pairwise comparisons between noise correlations of different FRA types performed on results of the above ANOVA, using Tukey’s HSD criterion.

| Compared FRA types | | Lower confidence interval | Mean | Upper confidence interval | p value |
| --- | --- | --- | --- | --- | --- |
| Single-peaked | Double-peaked | -0.0066 | -0.0066 | 0.0103 | 0.862 |
| Single-peaked | Complex | 0.0662 | 0.0788 | 0.0913 | 9.561x10^-10^ |
| Double-Peaked | Complex | 0.0629 | 0.0769 | 0.0910 | 9.561x10^-10^ |

**6. Figure 4C**

Unpaired t-test comparing the BF variation along the tonotopic gradient in single-peaked neurons and in double-peaked neurons in ferret.

| Group | Mean | Standard deviation | N (FRA) | t value | p value | Effect size  (Cohen’s d) |
| --- | --- | --- | --- | --- | --- | --- |
| Single-peaked | 0.755 | 0.746 | 296 | 2.425 | 0.016 | 0.216 |
| Double-peaked | 0.914 | 0.725 | 220 |  |  |  |

Unpaired t-test comparing the BF variation along the tonotopic gradient in single-peaked neurons and in complex neurons in ferret.

| Group | Mean | Standard deviation | N (FRA) | t value | p value | Effect size  (Cohen’s d) |
| --- | --- | --- | --- | --- | --- | --- |
| Single-peaked | 0.755 | 0.746 | 296 | 6.829 | 2.676x10^-11^ | 0.654 |
| Complex | 1.300 | 0.966 | 173 |  |  |  |

Unpaired t-test comparing the BF variation along the tonotopic gradient in double-peaked neurons and in complex neurons in ferret.

| Group | Mean | Standard deviation | N (FRA) | t value | p value | Effect size  (Cohen’s d) |
| --- | --- | --- | --- | --- | --- | --- |
| Double-peaked | 0.914 | 0.725 | 220 | 4.521 | 8.154x10^-6^ | 0.459 |
| Complex | 1.300 | 0.966 | 173 |  |  |  |

**7. Figure 5D**

Unpaired t-test comparing the local BF variation in single-peaked neurons and in double-peaked neurons in mice.

| Group | Mean | Standard deviation | N (FRA) | t value | p value | Effect size  (Cohen’s d) |
| --- | --- | --- | --- | --- | --- | --- |
| Single-peaked | 2.579 | 2.414 | 202 | 0.464 | 0.643 | -0.060 |
| Double-peaked | 2.737 | 3.148 | 87 |  |  |  |

Unpaired t-test comparing the local BF variation in single-peaked neurons and in complex neurons in mice.

| Group | Mean | Standard deviation | N (FRA) | t value | p value | Effect size  (Cohen’s d) |
| --- | --- | --- | --- | --- | --- | --- |
| Single-peaked | 2.579 | 2.414 | 202 | 4.337 | 2.029x10^-5^ | -0.584 |
| Complex | 4.148 | 3.313 | 76 |  |  |  |

Paired t-test comparing the local BF variation in double-peaked neurons and in the second peak of double-peaked neurons in mice.

| Group | Mean | Standard deviation | N (FRA) | t value | p value | Effect size  (Cohen’s d) |
| --- | --- | --- | --- | --- | --- | --- |
| Double-peaked | 2.737 | 3.148 | 87 | 1.149 | 0.254 | -0.140 |
| Peak2 | 3.194 | 3.369 | 87 |  |  |  |

**8. Figure 5E**

Unpaired t-test comparing the local BF variation in single-peaked neurons in ferrets and mice without accounting for ACx span and hearing range.

| Group | Mean | Standard deviation | N (FRA) | t value | p value | Effect size  (Cohen’s d) |
| --- | --- | --- | --- | --- | --- | --- |
| Ferret | 1.326 | 1.632 | 272 | 5.619 | 2.508x10^-8^ | -0.401 |
| Mice | 2.176 | 2.279 | 709 |  |  |  |

**9. Figure 5F**

2-way ANOVA comparing the proportion of BFs within expected range with FRA class and species as predictors.

| Dependent variable | Degrees of freedom | F value | p value | Effect size (Eta squared) |
| --- | --- | --- | --- | --- |
| FRA class | 2 | 8.41 | 4.3 x 10^-4^ | 0.14 |
| Species | 1 | 3.72 | 0.06 | 0.03 |
| Interaction | 2 | 1.66 | 0.19 | 0.03 |

Post-hoc pairwise comparisons between the proportion of BFs within expected range for different FRA types in mice relative to ferrets. Performed on results of the above ANOVA, using Tukey’s HSD criterion.

| FRA types compared between mice and ferrets | Lower confidence interval | Mean | Upper confidence interval | p value |
| --- | --- | --- | --- | --- |
| Single-peaked (BF_s_) | -0.31 | -6.7x10^-2^ | 0.18 | 0.97 |
| Double-peaked (BF_d_) | -0.47 | -0.22 | 3.2x10^-2^ | 0.12 |
| Complex (BF_d_) | -0.25 | -1.8x10^-3^ | 0.25 | 1.00 |
